# Effects of different continuous renal replacement therapy patterns on ionized calcium in patients with citrate anticoagulants using calcium-containing replacement solutions: a study protocol

**DOI:** 10.1080/07853890.2025.2523555

**Published:** 2025-06-25

**Authors:** Wan-Li Yan, Shu-Rong Gong, Rong-Guo Yu, Han Chen

**Affiliations:** a Fuzhou University Affiliated Provincial Hospital, Fujian Provincial Key Laboratory of Critical Care Medicine, Fujian Provincial Hospital, The Third Department of Critical Care Medicine, Fujian Shengli Clinical Medical College of Fujian Medical University, Fuzhou, China; b Fuzhou University Affiliated Provincial Hospital, Fujian Provincial Key Laboratory of Critical Care Medicine, Fujian Provincial Hospital, Cardiovascular Intensive Care Unit, Fujian Shengli Clinical Medical College of Fujian Medical University, Fuzhou, Fujian, China

**Keywords:** Acute kidney injury, continuous renal replacement therapy, regional citrate anticoagulation, calcium-containing replacement solutions

## Abstract

**Background:**

It is unclear whether different modes of continuous renal replacement therapy (CRRT) impact post-filter ionized calcium concentrations during regional citrate anticoagulation (RCA) when using calcium-containing replacement fluid.

**Methods:**

This prospective, single-center, observational cohort study will screen all patients receiving CRRT for eligibility. General clinical information will be collected before commencing CRRT treatment. Patients will be randomly assigned to either the continuous veno-venous hemofiltration (CVVH) or continuous veno-venous hemodialysis (CVVHD) group and switch to the alternative mode in the subsequent treatment session. Pre-filter and post-filter ionized calcium, systemic total and ionized calcium, and effluent total calcium will be measured 2 h after the initiation of CRRT. Electrolyte levels, arterial blood gases, hourly citrate dose, and total citrate dose will be recorded every 6 h until the end of CRRT. The primary outcome is the difference in ionized calcium concentrations at each site over time between the two modes.

**Discussion:**

This study will build upon clinical practice to explore the differential effects of various CRRT modes on ionized and total calcium in patients undergoing RCA-CRRT with calcium-containing replacement solutions.

## Introduction

Over the past decade, the prevalence of acute kidney injury (AKI) has increased significantly. The Kidney Disease Improving Global Outcomes (KDIGO) guidelines indicate that more than 50% of patients in the intensive care unit (ICU) have AKI, with approximately 10% of them requiring renal replacement therapy (RRT) [1,2]. Regional citrate anticoagulation (RCA) is widely used in continuous renal replacement therapy (CRRT) due to its low risk of bleeding and extended filter life [3–11].

Ionized calcium (iCa) is a crucial cofactor activating the coagulation cascade. Citrate acts as an anticoagulant by chelating iCa to form a stable calcium-citrate complex [12–14]. Post-filter iCa level is crucial for the adjustment of the citrate dose: the citrate dose is initially set based on an appropriate citrate dose/blood flow ratio and subsequently adjusted according to the post-filter iCa concentration to maintain a target iCa between 0.20 and 0.40 mmol/L in the circuit (equivalent to a blood citrate concentration of 3–4 mmol/L) in the clinical practice [15,16].

**Figure 1.**
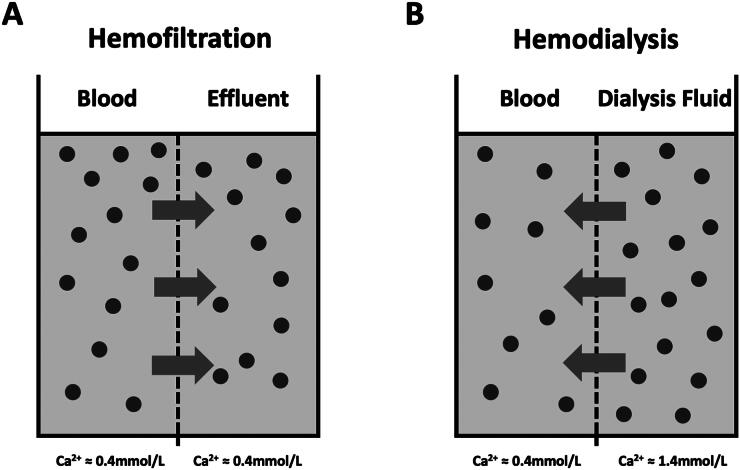
Theoretical trends of ionized calcium movement in different renal replacement therapies. Panel A: In hemofiltration, solute and solvent move from blood to effluent across a semipermeable membrane (dotted line) in response to transmembrane pressure. For citrate-anticoagulated blood, iCa concentration (solid dots) should be identical on both sides of the membrane, with a typical iCa of approximately 0.4 mmol/L. Panel B: In hemodialysis, the iCa concentration of calcium-containing dialysate (approximately 1.5 mmol/L) is much higher than the level of iCa in the citrate anticoagulated blood (approximately 0.4 mmol/L); therefore, iCa moves with the concentration gradient from the dialysate to the blood, resulting in an increase in post-filter iCa, which in turn may affect the efficacy of citrate anticoagulation.

Commercial calcium-containing replacement solutions are increasingly used in CRRT and have a higher iCa concentration than conventional replacement solutions [17–19]. Due to the different mechanisms of solute removal, there could theoretically be apparent differences in post-filter iCa and even systemic calcium when different CRRT modalities are used [20]. For example, in hemodialysis, solutes move with a concentration gradient, and a high concentration of iCa in the dialysate may gradually diffuse into the blood, increasing post-filter iCa (Figure 1A); in contrast, in hemofiltration, the solute and solvent move simultaneously with the pressure gradient, and the post-filter iCa concentration is unlikely to increase (Figure 1B). Since the post-filter iCa concentration is the basis for adjusting the dose of citrate, the potential difference in the post-filter iCa concentration under the different CRRT modalities using calcium-containing replacement solutions may lead to markedly different citrate dosages, even a different incidence of adverse events such as citrate accumulation and citrate toxicity [21–24].

However, the effect of citrate on post-filter iCa concentrations was investigated in the context of calcium-free replacement solutions in previous studies [25,26]. There is little data to show whether different CRRT modes can affect post-filter iCa concentrations during RCA-CRRT using calcium-containing replacement fluid. We hypothesize that a dialysis-based CRRT mode would increase post-filter iCa concentrations and citrate requirements. The present study aims to explore the influence of different CRRT modes on the post-filter iCa concentration in patients receiving RCA-CRRT using calcium-containing replacement fluid.

## Patients and methods

### Study design

This is a prospective, single-center, cross-over, observational cohort study in patients treated with RCA-CRRT. The study was registered at the ClinicalTrials.gov on 26 Aug 2023 (version 1.0, NCT06021080). The study protocol is reported according to the SPIRIT guidelines [27].

### Study setting and population

The study setting is the Surgical Intensive Care Unit (22 beds) of Fujian Provincial Hospital (2500 beds), Fujian Medical University, Fujian Province, China. All patients admitted to the ICU who need RCA-CRRT will be screened for study eligibility.

The inclusion criteria are 1) Age ≥ 18 years old; 2) Receiving citrate anticoagulation; 3) Informed consent obtained from patients or next of kin.

The exclusion criteria are 1) Pregnant or lactating women; 2) Allergic to citrate anticoagulants; 3) Severe liver dysfunction (total bilirubin levels exceeding two times the normal range); 4) Hypoxemia (PaO_2_ < 60 mmHg); 5) Inadequate tissue perfusion (blood pressure < 90/60 mmHg despite high doses of vasoactive agents); 6) Hyperlactatemia (lactate> 4 mmol/L); 7) Hypernatremia; 8) Estimated length of hospital stay < 48h; 9) Participated in other studies.

### Preparation of the replacement solution and citrate

A commercially available calcium-containing replacement solution will be used (Qingshan Licang Pharmaceutical Co, Chengdu, China, certification number: H20080452). When this product is used in combination with a 5% sodium bicarbonate injection (250 ml NaHCO_3_ for a 4000 ml bag), the concentration of each component is as follows: Na+ 141 mmol/L, Ca^2+^ 1.5 mmol/L, Mg^2+^ 0.75 mmol/L, Cl^-^ 110 mmol/L, Glucose 10.6 mmol/L, HCO_3_^−^ 35 mmol/L. During RCA-CRRT, the pre-blood pump infuses 4% trisodium citrate solution (Qingshan Licang Pharmaceutical Co, Chengdu, China, certification number: H20045612) into the extracorporeal circuit’s arterial line. In addition, T-branches will be connected at the end of the venous line, one for blood sampling and the other for connection to the sodium bicarbonate injection. To prevent the sodium bicarbonate from reacting with the calcium gluconate, the 10% calcium gluconate will be pumped into the body *via* a central venous line (peripherally inserted central catheter, subclavian catheterization, or internal jugular vein catheterization).

### CRRT parameter setting and management

PrismaFlex (Baxter Investment Co., Ltd, Chicago, IL) CRRT device and AN69 ST150 hemofilters (Baxter Investment Co., Ltd, Shanghai, China) will be used. Vascular access will be established by inserting a double-lumen catheter (SCW Medication Ltd, certification number: 20173664588, 12 Fr, 20 cm) into either the femoral or internal jugular vein. CRRT will be ended for any of the following reasons: achievement of therapeutic goals; coagulation of the filter; transmembrane pressure continuously > 250 mmHg; or continuous treatment for 48 h (to replace pipeline and filter).

This study is a before-after design in the same patient; each patient will receive both continuous veno-venous hemofiltration (CVVH) and continuous veno-venous hemodialysis (CVVHD) at least once. Patients will be initially randomized into either the CVVHD or CVVH groups and then crossed over to the other group during the second CRRT session. Regardless of whether CVVH or CVVHD is used, the initial settings will be as follows: blood flow rate of 150 ml/min, 4% sodium citrate flow rate of 230 ml/h, 10% calcium gluconate infusion rate of 10 ml/h, and a 5% sodium bicarbonate infusion rate (ml/h) of dialysate/replacement fluid × 0.0625 − 120. The therapeutic dose will be 30 ml/kg/h depending on the mode investigated, that is, a dialysate rate (ml/h) of 30 ml × body weight (kg) in the setting of CVVHD; on the other hand, the replacement fluid rate (ml/h) will be 30 ml × body weight (kg) in the setting of CVVH.

The clinician will assess whether to adjust the CRRT parameters based on arterial blood gas and post-filter iCa concentration two hours after the start of CRRT. No adjustment of the citrate dose is required, and post-filter iCa levels will be monitored every four to six hours if the therapeutic goal of anticoagulation is achieved (post-filter iCa concentration 0.20–0.4 mmol/L). If anticoagulation is not achieved, the citrate infusion rate will be adjusted according to Table 1, and post-filter iCa levels will be monitored every two hours until the therapeutic goal is achieved. The goal of systemic iCa will be between 1.00 and 1.20 mmol/L. The 10% calcium gluconate infusion rate will not be adjusted, and arterial blood gas will be monitored every four to six hours if serum iCa concentration reaches the target. The calcium gluconate infusion rate will be adjusted according to Table 2, and arterial blood gas will be monitored every two hours until the target is reached if serum iCa concentration does not reach the target.

**Table 1.** Adjustment of 4% sodium citrate infusion rate according to post-filter ionized calcium level.

Post-filter iCa (mmol/L)	Change in citrate infusion rate
<0.2	20ml/h ↓
0.2–0.4	No change
0.41–0.45	10ml/h ↑
>0.45	20ml/h ↑

**Table 2.** Adjustment of calcium gluconate infusion rate according to systemic ionized calcium level.

Systemic ionized Ca^2+^ (mmol/L)	Change in 10% calcium gluconate infusion rate
>1.35	5ml/h ↓
1.2–1.35	2ml/h ↓
1.0–1.2	No change
0.91–0.99	2ml/h ↑
0.86–0.90	5ml/h ↑
0.75–0.85	7.5 ml/h ↑
<0.75	10ml/h ↑

If circuit clotting is detected, the circuit will be discontinued immediately to ensure patient safety. All clotting events will be documented in detail, including the time of detection, last recorded post-filter iCa level, circuit running time before clotting, and citrate dose at the time of clotting. The attending physician will then evaluate the patient’s clinical condition to determine whether immediate CRRT re-initiation is necessary or if CRRT can be temporarily discontinued until the next scheduled treatment. If clinical assessment indicates the need for immediate CRRT continuation, a new circuit will be initiated following the standard protocol. Given the short half-life of citrate, the time required for circuit replacement is believed to be sufficient to eliminate any carry-over effects.

### Data collection and follow-up

**Figure 2.**
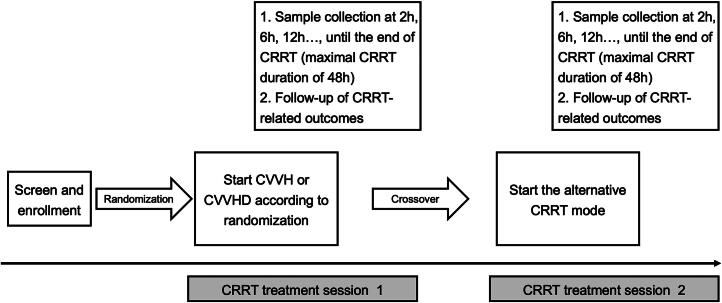
Flowchart of the study. CRRT: continuous renal replacement therapy; CVVHD: continuous veno-venous hemodialysis; CVVH: continuous veno-venous hemofiltration;

Figure 2 illustrates the process of the entire study. Clinical information will be collected prior to the initiation of CRRT. This includes gender, age, weight, baseline blood urea nitrogen, serum creatinine, urine output, comorbidities, Acute Physiology and Chronic Health Evaluation (APACHE)-II scores, Sequential Organ Failure Assessment (SOFA) score, hemoglobin, platelets, serum total calcium, and ionized calcium. Renal function will be classified according to KDIGO criteria [2].

**Figure 3.**
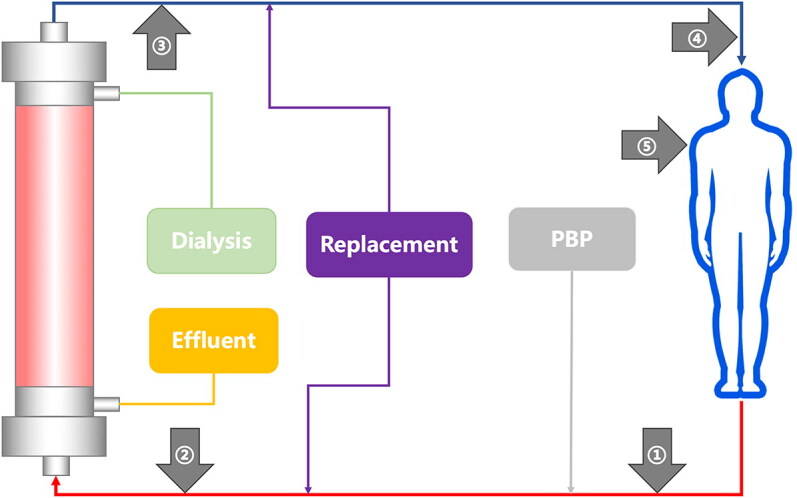
Blood sample collection sites at various locations during continuous renal replacement therapy. ①: pre-filter iCa ②: post-filter iCa ③: systemic total and ionized calcium ④: effluent total calcium PBP: pre-blood pump; iCa: Ionized calcium

Pre-filter iCa, post-filter iCa, systemic total calcium, ionized calcium, and effluent total calcium will be measured two hours after the start of CRRT for each new circuit. The sites of blood sample collection are shown in Figure 3. The measurements will be performed using an arterial blood gas analyzer for serum ionized calcium, post-filter iCa, and pre-filter iCa. Blood samples will be sent to the central laboratory to measure systemic total calcium. Effluent total calcium will be measured by inductively coupled plasma emission spectrometry.

Electrolytes, arterial blood gas analysis, hourly citrate dose, and total citrate dose will be recorded every 6 h until the end of CRRT. The concentration of the 4% sodium citrate solution is 136 mmol/L, and the screening factor for citrate is 1.0. Therefore, the average citrate dose entering the patient’s body per hour will be calculated as follows: average citrate dose = 136 mmol/L * citrate infusion rate (L/h) * (1 - ultrafiltration rate/plasma flow). The total citrate dose will be calculated as follows: total citrate = hourly citrate dose * total treatment time.

At the end of CRRT, filter and venous pot clotting and individual filter lifetime will be recorded. Filter lifetime is defined as the time (in hours) from the start of CRRT to clotting or the end of the procedure. All data will be prospectively collected by physicians who are not involved in patient care.

To control for potential confounding factors from blood products containing citrate, the following measures will be implemented: 1) Blood products will only be administered when clinically necessary. 2) The timing and volume of all blood product administrations will be carefully documented. 3) Additional post-filter iCa levels will be measured at 2 and 4 h after any blood product administration. 4) These events will be recorded as potential confounding factors and will be considered in the final analysis.

### Study objectives

The primary objective is to evaluate trends in post-filter iCa2+, pre-filter iCa effluent iCa2+ and total calcium, systemic iCa2+, and total calcium concentration over time in different RCA-CRRT modes.

The secondary objectives are to analyze the amount of citrate used in different modes to achieve the desired anticoagulant effect in current clinical practice, and to assess the incidence of new-onset metabolic complications (hypercalcemia, hypocalcemia, metabolic acidosis, metabolic alkalosis, citrate accumulation, hypernatremia, hyponatremia). The incidence of metabolic complications will be calculated as the number of new-onset metabolic complication events per 100 CRRT days. In this study, citrate accumulation is defined as the presence of a total serum calcium/ionized calcium ratio > 2.5.

### Sample size

According to the results of our pilot study, the post-filter iCa concentration was 0.44 mmol/L in the CVVHD mode and 0.305 mmol/L in the CVVH mode. Assuming a standard deviation of 0.2 mmol/L, taking a *α* = 0.05 and a *β* = 0.10, the required sample size is 26 cases. Thirty patients will be included in the study considering the possible loss of follow-up.

### Statistical analysis

Data will be analyzed using SPSS 23.0 statistical software (IBM Co., NY). Continuous variables will be presented as means and standard deviations (for normal distribution) or medians and interquartile ranges (for non-normal distribution). A paired *t*-test will be used for normally distributed variables, and the Wilcoxon signed-rank test will be used for non-normally distributed variables. Categorical variables will be presented as numbers and percentages and analyzed using the *χ*^2^-test or Fisher’s exact tests, as appropriate. For the crossover design, we will specifically examine both carry-over and period effects. The carry-over effect will be assessed by comparing the sum of the outcomes between sequences using a t-test or Mann-Whitney U test depending on data distribution. The sequence of interventions will be randomly assigned to minimize potential period effects. A two-sided *p* < 0.05 will be considered a statistically significant difference.

### Randomization, blind, and allocation concealment

The study is a prospective, randomized, crossover-designed study. Random assignment is based on a computer-generated random number table using a concealed sealed and numbered envelope procedure to assign patients to one of the two study groups prior to the first CRRT mode. Treatment allocation is blinded to patients and other investigators, with the exception of bedside clinicians and nurses. Investigators who perform the blood gas analysis are unaware of the details of the trials.

### Data management

The research data will be accessible only to members of the research team. All data collected will be recorded in the patient’s file and securely stored in a locker. After the completion of the study, all case report forms will be archived. Electronic files will be password-protected to ensure confidentiality. Both paper and electronic files will be retained per the hospital’s policy on the retention and disposal of patient records.

## Discussion

Post-filter iCa level is crucial for adjusting the citrate dose during RCA-CRRT. However, there is a lack of clinical data demonstrating the effects of different CRRT modes on ionized and total serum calcium levels in patients receiving RCA-CRRT with a calcium-containing replacement solution. We hypothesize that post-filter iCa concentration is higher in CVVHD than in CVVH under RCA-CRRT using a calcium-containing replacement solution. If this hypothesis is true, patients may require a higher citrate dose, thereby increasing the risk of citrate-related adverse effects. This study will expand previous work and provide new data regarding the impact of different RCA-CRRT modes on iCa, citrate dose, and citrate-related adverse effects.

While directly measuring citrate and calcium concentrations in the ultrafiltrate would provide a more controlled assessment of solute removal across different CRRT modes, our study aims to evaluate the clinical implications of citrate removal in actual patient care settings. The patient-based approach allows us to observe how variations in citrate removal affect circuit survival and safety parameters under real clinical conditions, including the influence of individual patient factors. This design better reflects the practical challenges and outcomes in routine clinical practice, though we acknowledge it introduces more variability in our measurements. The trade-off between controlled laboratory conditions and clinical relevance was carefully considered in our study design, and we chose to prioritize clinical applicability of our findings.

The iCa levels can be influenced by multiple factors including blood pH, acid-base status, and phosphate levels. To consider these potential confounding factors, we will collect comprehensive blood gas analysis data and biochemical parameters throughout the CRRT sessions. The crossover design of our study will help minimize between-subject variations, as each patient will serve as their own control. Furthermore, we will include these parameters as covariates in our statistical analysis to adjust for their potential effects on iCa levels. This comprehensive analytical approach will strengthen the reliability of our conclusions despite the complex nature of factors affecting iCa homeostasis.

There are several limitations to this study. First, it will only compare the differences in iCa changes between CVVH and CVVHD at each time point, thus not providing information on iCa concentration changes in combined modes (e.g. continuous veno-venous hemodiafiltration, CVVHDF) with varying dialysate and replacement fluid ratios. In other words, this study focuses exclusively on CVVH and CVVHD modes. While CVVHDF is increasingly used in severe AKI patients, we chose CVVH and CVVHD as our initial investigation platforms to establish the fundamental principles in more controlled settings. This strategic approach will serve as a stepping stone for future studies investigating more complex CRRT modalities such as CVVHDF. Second, plasma citrate concentrations will not be measured in this study, making it impossible to directly compare differences in plasma citrate concentrations.

## Ethics and dissemination

### Ethical approval and informed consent

The study protocol and consent forms were approved on 7 July 2023 by the Ethics Committee of Fujian Provincial Hospital (Approval #K2023-07-007). Protocol amendments (if any) will be approved by the Ethics Committee.

Following the screening process, a designated study coordinator will be introduced to the family by the ICU physician. The study coordinator’s credentials will be clearly communicated to the family to establish trust and credibility. The study coordinator will provide a comprehensive explanation of all relevant aspects of the project, pausing frequently to address any questions or concerns the family may have. In addition, the coordinator will ask the family to summarize the information discussed in their own words to ensure understanding.

During the discussion, it will be emphasized that participation in the study is completely voluntary and that the family has the right to refuse consent without any consequences. The coordinator will also emphasize that the family can withdraw their consent at any time during the study. To ensure proper documentation, written consent will be obtained in the presence of a witness.

### Dissemination of results

No interim analysis is planned for this study. The results will be submitted to an international peer-reviewed journal for publication. Additionally, the findings will be shared through national and international scientific conferences to disseminate the research to a wider audience. The corresponding authors will make the data sets from this study available to interested parties upon a reasonable request.

## Supplementary Material

SPIRIT_Checklist.doc

## Data Availability

No data were generated in this work as this is a study protocol.
